# Management of Heparin-Induced Thrombocytopenia with Fondaparinux in a Patient with Left Ventricular Assist Device

**Published:** 2014-05-01

**Authors:** V. Velagic, J. Samardzic, Z. Baricevic, B. Skoric, M. Cikes, H. Gasparovic, B. Biocina, D. Milicic

**Affiliations:** 1 *Department for Cardiovascular Diseases, University Hospital Centre Zagreb, Croatia*; 2 *University of Zagreb School of Medicine, Zagreb, Croatia*; 3 *Department of Cardiac Surgery, University Hospital Centre Zagreb, Croatia*

**Keywords:** Heart failure, Heparin-induced thrombocytopenia, Sepsis, Ventricular assist device, Anticoagulation

## Abstract

Heparin-induced thrombocytopenia is an immune-mediated serious adverse effect of heparin therapy. It is a relatively frequent complication among patients with mechanical circulatory support. Herein, we present a patient with severe heart failure and sepsis who developed heparin-induced thrombocytopenia shortly after implantation of left ventricular assist device as a bridge to transplantation and who was successfully treated with fondaparinux.

## INTRODUCTION

Heparin-induced thrombocytopenia is an immune-mediated serious adverse effect of heparin therapy caused by IgG antibodies against heparin/platelet factor 4 (PF4) complexes, resulting in thrombocytopenia associated with thromboembolic events. Heparin-induced thrombocytopenia is a relatively frequent complication among patients with mechanical circulatory support and is associated with unfavorable outcome [1]. Currently, only direct thrombin inhibitors (bivalirudin, argatroban, lepirudin) are approved by the Food and Drug Administration for the treatment of heparin-induced thrombocytopenia [2]. Herein, we present a patient who developed heparin-induced thrombocytopenia shortly after implantation of a left ventricular assist device (LVAD, Levitronix CentriMag) who was successfully treated with fondaparinux, a synthetic pentasaccharide factor Xa inhibitor.

## CASE REPORT

A 44-year-old man was admitted to our hospital with congestive heart failure. He had been treated with optimal medical therapy (*ie*, beta-blocker, ACE-inhibitor, spironolactone, loop diuretic, amiodarone, warfarin), but the treatment was not successful. Oral anticoagulant therapy was introduced because of severe systolic dysfunction. The patient had a long history of idiopathic dilated cardiomyopathy and frequent hospitalizations due to recurrent acute cardiac decompensation. The patient had no other co-morbidities besides subclinical hypothyroidism. Unlike previous episodes of acute decompensation, when the patient presented to our center with volume overload, signs of low cardiac output became predominant clinical features. On the second day of hospitalization, warfarin was switched to enoxaparin because of presumed invasive procedures. Echocardiographic examination revealed a dilated left ventricle (diameter of 7.56 cm) with severely impaired systolic function (ejection fraction of 20%), restrictive diastolic dysfunction and severe mitral regurgitation. Electrocardiography showed sinus tachycardia (rate of 100–130 beats/min) with left anterior hemiblock. The patient developed cardiogenic shock, which required intravenous inotropic support. Over the next few days, he developed sepsis (temperature up to 38.5 ºC, C-reactive protein 140 g/L, lymphocyte count of 29×10^9^/L with shift to the left) without focal signs of infection. There was no infiltration on chest roentgenogram; repeated urine and blood cultures were sterile. 

We presumed that low output syndrome and visceral hypoperfusion resulted with bacterial transudation from the gastrointestinal tract. We therefore administered metronidazole and gentamicin. However, further hemodynamic deterioration with acute kidney and liver failure occurred. We started dopamine and then noradrenaline support. Continuous veno-venous hemodialysis was also performed due to renal failure. The patient responded markedly to antibiotics and became afebrile after two days. Inflammatory markers decreased and his hemodynamic condition became partially stabilized. Kidney and liver function improved, though he further needed inotropic and vasopressor support with dobutamine and dopamine. Swan-Ganz catheterization showed very low cardiac output (2.56 L/min) and cardiac index (1.12 L/min/m2). We concluded that our patient needed a mechanical heart support as a bridge to heart transplantation. 

He was transferred to the operating room and a left ventricular assist device was implanted. The procedure was uneventful and the patient was anticoagulated with standard unfractionated heparin. On the fourth post-operative day the patient complained of sudden shortness of breath without signs of pulmonary congestion. The central venous pressure increased from 18 to 36 mm Hg; the pulmonary artery pressure increased from 45/30 to 70/50 mm Hg. Echocardiographic examination showed a severely dilated and hypocontractile right ventricle. The platelet count decreased from 190×10^9^/L to 80×10^9^/L; D-dimers value was 3 μg/mL. Heparin-induced thrombocytopenia with pulmonary embolism was suspected. We could not perform pulmonary angiography or scintigraphy to confirm the diagnosis, due to technical difficulties. Unfractionated heparin was discontinued and blood sample was drawn for enzyme-linked immunosorbent assay (ELISA) and micro-typing system assays. For lack of other approved anticoagulant drugs for heparin-induced thrombocytopenia in our hospital, fondaparinux (Arixtra) was administered. 

Because the need for anticoagulant therapy was urgent, no medical ethical approval was obtained. The patient was well informed of the situation and signed a consent form to take fondaparinux.

**Figure 1:**
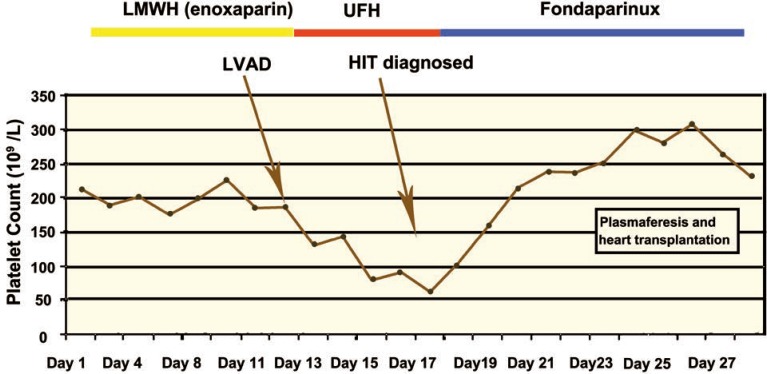
Platelet count and anticoagulant therapy schedule in the patient

We started with initial dose of 7.5 mg of fondaparinux; further dose adjustment was performed according to anti-Xa assay (target levels 0.6–1 U/mL). Both ELISA and micro-typing system assays were strongly positive for heparin-induced thrombocytopenia. In the next few days after discontinuation of unfractionated heparin, the patient’s clinical status improved and platelet count returned back to normal (Fig 1). While on fondaparinux, the left ventricular assist device did its job with no complications; there was no increase in D-dimer or hemolysis. At the time of fondaparinux introduction to therapy, the patient’s creatinine was 119 µmol/L; his glomerular filtration rate (GFR) was estimated at 61 mL/min/1.73 m^2^. 

The patient was placed on the Eurotransplant waiting list for heart transplantation and granted the high urgent status. A donor heart was found after 14 days on mechanical circulatory support. Just prior to the operation, plasmapheresis was performed and successful orthotopic heart transplantation was completed using unfractionated heparin. Post-transplantation period followed without any complications. Fondaparinux was discontinued after total mobilization of the patient on the 24^th^ post-operative day. His mean platelet level after transplantation and before discharging from the hospital was 177.8×10^9^/L (range: 109–231). During fondaparinux therapy, the patient serum creatinine remained normal; the estimated GFR improved. The patient was discharged from the hospital on the 26^th^ post-operative day in good clinical condition.

## DISCUSSION

Thrombocytopenia is a common laboratory finding in critically ill patients. Sepsis and hemodilution are two common causes of thrombocytopenia, however, heparin-induced thrombocytopenia should also be considered in the differential diagnosis list [3]. Heparin-induced thrombocytopenia usually develops 5–10 days after the introduction of heparin [1]. It is associated with platelet activation and aggregation, endothelial activation, and thrombin generation with consequential thromboembolic incidents and thrombocytopenia. Therefore, platelet count should be regularly monitored in patients receiving heparin. The thrombocytopenia occurs in approximately 3% of those receiving unfractionated heparin and in less than 1% of those receiving low-molecular weight heparin [4]. If heparin-induced thrombocytopenia is suspected, all heparin products should be discontinued and replaced with an alternative rapid-acting anticoagulant. Currently, direct thrombin inhibitors (*eg*, argatroban, lepirudin, bivalirudin) are approved by the FDA for the management of heparin-induced thrombocytopenia. These drugs have chemical structures different from unfractionated and low-molecular weight heparin and do not cross-react with the produced antibodies. Some authors hypothesize that fondaparinux, which is a selective factor Xa inhibitor with a structure of a pentasaccharide chain, is too short to induce an antibody response and thus could be useful for treating heparin-induced thrombocytopenia. 

Fondaparinux is not currently approved for the treatment of heparin-induced thrombocytopenia; however, it has been used in anecdotal case reports [5]. On the other hand, there are reports that heparin-induced thrombocytopenia could also be related with fondaparinux [6, 7]. The development of heparin-induced thrombocytopenia is a major clinical problem and is associated with unfavorable outcomes in patients on mechanical circulatory support [1]. Arterial and venous thromboembolism complicate as many as 50% of these cases, and carry 20%–30% mortality risk. In accordance with its onset, heparin-induced thrombocytopenia has different presentations: a) “classic” or typical onset of heparin-induced thrombocytopenia with a relative decrease in platelet count for ≥50% or absolute decrease with <150×10^9^/L within 4–14 days of heparin therapy, b) rapid onset of heparin-induced thrombocytopenia, if the patient has antibodies from previous heparin exposure (within the past 100 days) when the drop in platelet count may begin within 24 hrs after heparin initiation, and c) delayed onset of heparin-induced thrombocytopenia that occurs several days or sometimes up to three weeks after heparin has been discontinued [8]. Despite the lack of confirmation by a serotonin-release functional assay, the “gold standard” method with the highest specificity, the diagnosis of heparin-induced thrombocytopenia in our patient was based on strong clinical and laboratory grounds—the patient had a significant drop in platelet count four days after introduction of unfractionated heparin (absolute platelet count <150×10^9^/L and >50% decrease from basal value); he developed a thromboembolic event (clinical manifestations of pulmonary embolism); he had two different positive heparin-induced thrombocytopenia serology assays; and his platelet count completely recovered after discontinuation of heparin. Since cardiac transplantation mandates the use of unfractionated heparin (at the time, other options were not available in our institution) there was a question of heparin re-exposure. There is published evidence that peri-operative plasmapheresis can be used in cardiac surgery patients who have a recent history of heparin-induced thrombocytopenia [9]. We used this approach without further complications. Our patient was treated with fondaparinux therapy for 10 days and had no complications with mechanical circulatory support during this period. 

This case demonstrates that fondaparinux might be used as an alternative for argatroban, lepirudin, bivalirudin, in patients with heparin-induced thrombocytopenia.

To our knowledge, this is the second case report on successful use of fondaparinux in a patient with heparin-induced thrombocytopenia on ventricular assist device [10]. Our case showed that fondaparinux could be successfully used even in high risk patients with huge thrombotic burden and numerous complications related to multiple organ failure and severe sepsis. 
